# In reply: espying sarcopenia in gastric cancer: squaring the circle

**DOI:** 10.1093/oncolo/oyae232

**Published:** 2024-09-03

**Authors:** Wing-Lok Chan

**Affiliations:** Department of Clinical Oncology, School of Clinical Medicine, LKS Faculty of Medicine, The University of Hong Kong, Hong Kong, Hong Kong SAR, China

## Abstract

This letter to the editor continues the discussion on sarcopenia in gastric cancer.

Thank you for your insightful feedback^1^ on our recent publication examining the association between sarcopenia, measured by skeletal muscle index (SMI) on computed tomography (CT), and clinical outcomes in patients with advanced gastric cancer. In our study, we found that prechemotherapy SMI cut-off values of ≤33 cm^2^/m^2^ for males and ≤ 28 cm^2^/m^2^ for females were able to identify patients with advanced gastric cancer who were at higher risk of experiencing severe chemotherapy toxicities and had a worse prognosis.^2^ These findings underscore the clinical relevance of accurately assessing skeletal muscle status in this patient population.

Sarcopenia is a complex geriatric syndrome characterized by the progressive and generalized loss of skeletal muscle mass and function.^3,4^ The diagnosis of sarcopenia, as defined by the European Working Group on Sarcopenia in Older People, requires the presence of both low muscle mass and low muscle strength or physical performance. Accurately identifying sarcopenia is crucial, as it is associated with adverse clinical outcomes, including increased risk of falls, functional decline, and mortality.

Muscle ultrasound is an emerging tool for the assessment of sarcopenia.^5,6^ It has several advantages, such as providing real-time, dynamic evaluation of muscle size, structure, and function, as well as being easily accessible, radiation-free, and portable compared to CT imaging.

However, there are also limitations to the use of ultrasound for assessing sarcopenia. These include the need for specialized probes to achieve varying depth and resolution, limited utility in obese patients, user-dependence in image quality and interpretation, lack of clear and standardized protocols, and absence of established cutoff values for diagnosing low muscle mass.^7^ Moreover, a systematic review on the diagnostic accuracy of ultrasound for sarcopenia diagnosis found it had a low-to-moderate accuracy for sarcopenia on the quadriceps muscle, with an AUC of 0.67-0.71^6^.

Despite the potential of muscle ultrasound, using MRI or CT imaging remain the gold standard for evaluating muscle mass in clinical research and practice.^8^ This is particularly relevant in our study of patients with advanced gastric cancer, where CT scans are routinely performed as part of standard clinical care, providing readily available and reliable data on skeletal muscle status. Furthermore, the use of CT-derived SMI has been extensively investigated, with well-established cut-off values for the diagnosis of low muscle mass and its association with clinical outcomes, including survival and chemotherapy toxicity.^2,9,10^ To replace CT with ultrasound for skeletal muscle evaluation, rigorous comparisons between these 2 techniques, along with the development of normative data and standardized protocols, are essential to validate the reliability and reproducibility of muscle ultrasound.

As the assessment of sarcopenia continues to evolve, future research should aim to incorporate comprehensive evaluation of skeletal muscle status and function. Combining biomarkers and clinical parameters beyond just muscle mass may determine sarcopenia’s utility in predicting outcomes.^11^ Exploring multimodal imaging, including CT and emerging ultrasound, could provide deeper insights. Establishing universal SMI cut-offs and standardized protocols for specific cancer types are crucial next steps to enable reliable, comparable assessment, and inform clinical decisions. These advancements would enhance the applicability and comparability of sarcopenia research, leading to more informed management and improved patient outcomes, especially in the older population with cancer.
